# Breast Cancer-Derived Exosomes Alter Macrophage Polarization *via* gp130/STAT3 Signaling

**DOI:** 10.3389/fimmu.2018.00871

**Published:** 2018-05-08

**Authors:** Sunyoung Ham, Luize G. Lima, Edna Pei Zhi Chai, Alexandra Muller, Richard J. Lobb, Sophie Krumeich, Shu Wen Wen, Adrian P. Wiegmans, Andreas Möller

**Affiliations:** ^1^Tumour Microenvironment Laboratory, QIMR Berghofer Medical Research Institute, Brisbane, QLD, Australia; ^2^Faculty of Health, School of Biomedical Sciences, Queensland University of Technology, Brisbane, QLD, Australia; ^3^Faculty of Medicine, University of Queensland, Brisbane, QLD, Australia; ^4^Faculty of Medical Biology, University Duisburg-Essen, Essen, Germany; ^5^Centre for Inflammatory Diseases, Department of Medicine, School of Clinical Sciences, Monash University, Clayton, VIC, Australia

**Keywords:** cancer-derived exosomes, breast cancer, tumor-associated macrophages, glycoprotein 130, interleukin-6, STAT3

## Abstract

Tumor-derived exosomes are being recognized as essential mediators of intercellular communication between cancer and immune cells. It is well established that bone marrow-derived macrophages (BMDMs) take up tumor-derived exosomes. However, the functional impact of these exosomes on macrophage phenotypes is controversial and not well studied. Here, we show that breast cancer-derived exosomes alter the phenotype of macrophages through the interleukin-6 (IL-6) receptor beta (glycoprotein 130, gp130)-STAT3 signaling pathway. Addition of breast cancer-derived exosomes to macrophages results in the activation of the IL-6 response pathway, including phosphorylation of the key downstream transcription factor STAT3. Exosomal gp130, which is highly enriched in cancer exosomes, triggers the secretion of IL-6 from BMDMs. Moreover, the exposure of BMDMs to cancer-derived exosomes triggers changes from a conventional toward a polarized phenotype often observed in tumor-associated macrophages. All of these effects can be inhibited through the addition of a gp130 inhibitor to cancer-derived exosomes or by blocking BMDMs exosome uptake. Collectively, this work demonstrates that breast cancer-derived exosomes are capable of inducing IL-6 secretion and a pro-survival phenotype in macrophages, partially *via* gp130/STAT3 signaling.

## Introduction

Breast cancer is the second most frequently diagnosed cancer type for females worldwide, accounting for approximately 1.67 million cases per year (1). The primary cause of mortality in breast cancer patients is caused by the spread of tumor cells to other organs such as lung (2), brain (3), and bone (4). Recently, small vesicles released by cancerous cells, termed as exosomes, were described to be markers, mediators, and inducers of metastasis (5).

Exosomes are small extracellular, bilipid vesicles that exhibit a size distribution of 30–150 nm, sediments at 100,000 *g*, and have a specific density of 1.13–1.19 g/mL (6–8). In contrast to other vesicles, exosomes are secreted after fusion of multivesicular bodies with the plasma membrane, resulting in proteins involved in this process to be uniquely associated with exosomes (9). Associated proteins include parts of the endosomal sorting complex required for transport proteins (Hrs and Tsg101) and tetraspanins (CD9, CD63, and CD81) (9). Exosomes derived from diverse types of cancer cells, including leukemia, ovarian, lung, and breast cancer, have shown distinct molecular profiles when compared with exosomes produced by corresponding untransformed, normal cells (9, 10).

Previous work from our group showed that breast cancer-derived exosomes accumulate in the lung, spleen, and bone of naïve mice (11). At these sites, the exosomal content causes pro-metastatic alterations associated with reduction of both T cell proliferation and NK cell cytotoxicity (11). For this reason, exosomes have become a valuable target in identifying novel cancer biomarkers that could potentially diagnose cancer and predict patient outcomes or treatment responses (12, 13).

Even though their role in immune response modulation is not completely understood, recent studies have shown that cancer-derived exosomes can direct immune cells toward a tumor-promoting phenotype, and significantly contribute to different aspects of tumor progression, including promotion of tumorigenesis, invasion of the surrounding tissues, angiogenesis, formation of pre-metastatic niches, and metastatic dissemination (14). For example, tumor-derived exosomes regulate the differentiation of myeloid progenitor cells (15). Furthermore, breast carcinoma-derived exosomes have been demonstrated to mediate the recruitment of myeloid-derived cells to the spleen and tumor, which in turn promotes cancer growth and neo-angiogenesis (16). Classically activated macrophages can respond to cancer cells with phagocytosis and release of inflammatory cytokines triggered by tumor-associated antigens. On the other hand, macrophages infiltrating established tumors are known to produce anti-inflammatory cytokines and support tumor progression (17). These cancer-associated macrophages have also been demonstrated to contribute to metastasis, especially to the formation of the pre-metastatic niche (16). For example, STAT3 phosphorylation, and therefore activation, in macrophages is commonly observed in the tumor microenvironment. Blockade of STAT3 signaling in these cells results in the secretion of pro-inflammatory cytokines (18, 19).

Interleukin-6 (IL-6) is regarded as both as a pro- and anti-inflammatory cytokine. Upon activation of IL-6 signaling, IL-6 receptors, such as IL-6Rα and IL-6Rβ (also known gp130), engage to form a dimeric structure (20). Signaling *via* these receptors activates JAK tyrosine kinases and transcriptional factors, in particular, STAT3 (21). Once STAT3 is activated, it translocates into the nucleus, inducing gene expression of IL-6, LOX, and other genes, creating an induction of IL-6 autocrine loop and tumorigenesis (22). Macrophages activated by interferon gamma (IFNγ) and LPS express high levels of IL-6 (23). There is evidence that IL-6 is also expressed by macrophages found in the tumor microenvironment, especially by alternatively polarized macrophages (24, 25). Furthermore, it has been reported that blockade of IL-6 affects tumor-infiltrating immune subsets, for example, reducing the number of myeloid-derived suppressor cells and their suppressive abilities (26). This is also observed in the development of lung cancer, with reduced frequency of tumor-associated macrophages which produce Arg1, CCL2, IL-10, and TGF-β (26).

This study reveals that IL-6 receptor gp130 is contained in breast cancer cell-derived exosomes and stimulates STAT3 signaling in bone marrow-derived macrophages (BMDMs). In response to exosome exposure, these BMDMs upregulate pro- and anti-inflammatory cytokines and acquire an increased survival potential. Our findings indicate that cancer-derived exosomes are capable of changing macrophage phenotype by transferring the IL-6 receptor gp130, thereby assisting in establishing a pro-tumorigenic cancer microenvironment.

## Materials and Methods

### Mice

C57Bl/6 mice were purchased from the Walter and Eliza Hall Institute (Melbourne, VIC, Australia) and female mice used at 8–10 weeks of age. All animal procedures were conducted in accordance with Australian National Health and Medical Research regulations on the use and care of experimental animals, and approved by the QIMR Berghofer Medical Research Institute Animal Ethics Committee (A12617M, P1499).

### Cell Culture

The murine C57BL/6 EO771 cells were maintained in DMEM with 5% FBS and 1% penicillin/streptomycin as described previously (27).

### Antibodies and Reagents

Synthetic unilamellar 100-nm sized liposomes (nanoparticles made from phosphatidylcholine and cholesterol, but lacking any protein content) were purchased from Encapsula Nanosciences. The primary and secondary antibodies used for western blotting, immunofluorescence, and flow cytometry are as listed in Table S1 in Supplementary Material.

### Isolation of Exosomes

The culture supernatants of EO771 cells at approximately 60–70% confluence were harvested after 16 h conditioning in serum-free media (11). Exosomes were isolated as previously described (6). Briefly, cells and debris were cleared from the supernatant by centrifugation (500 *g*, 10 min), followed by filtration using 0.22 μm filters (Merck Millipore). Cell-free supernatants were concentrated by ultrafiltration through Centricon Plus-70 Centrifugal Filter (100 kDa; Merck Millipore), spun at 3,500 *g* at 4°C. Exosomes were subsequently purified by overlaying concentrated samples on qEV size exclusion chromatography columns (Izon Science Ltd.) followed by elution with PBS. Finally, the elute from qEV columns were concentrated using Amicon Ultra-4 10-kDa nominal molecular weight centrifugal filter units (Merck Millipore) to a final volume of approximately 200 µL.

### Size Distribution Analysis by Tunable Resistive Pulse Sensing (TRPS)

Particle abundance and size were assessed using the Izon qNano system by TRPS technology (Izon Science Ltd.) with the NP100 nanopore and 70-nm calibration beads (CPS70) as previously reported (11).

### Electron Microscopy (EM)

Electron microscopy imaging was performed as previously described (6). Briefly, purified exosomes were fixed with paraformaldehyde and transferred to Formvar-carbon-coated EM grids. Grids were transferred to 1% glutaraldehyde for 5 min, followed by eight washes with water. By contrast, grids were negatively stained with 1% uranyl-oxalate solution, pH 7 for 5 min before transferring to methyl-cellulose-UA for 10 min. Excess fluid was removed and exosomes were imaged in a JEOL 1011 transmission electron microscope at 60 kV.

### Western Blotting

Exosome preparations and cell lysates were solubilized with RIPA buffer. Protein content was quantified using a standard Bradford assay or a BCA assay and analyzed by western blotting as previously described (28). Each western was independently repeated at least three times, and representative results are shown. Full-length images of all western results shown in the manuscript are included as Figure S5 in Supplementary Material.

### DiD Labeling of Exosomes

Exosomes were fluorescently labeled using Vybrant^®^ DiD (Life Technologies) according to the manufacturer’s instructions with modifications (11). Briefly, exosomes were incubated for 10 min with DiD (1:1,000 dilution in PBS) at room temperature and re-purified using qEV size exclusion chromatography columns (Izon Science Ltd.).

### Generation of BMDMs

Bone marrow cells were obtained by flushing the femurs and tibias of C57Bl/6 mice. Cell suspensions were treated with ammonium chloride red cell-lysis buffer, washed with PBS, and then 4 × 10^5^ cells/well were cultured in 6-well plates in RPMI supplemented with L cell conditional media (10% FBS, 1% GlutaMAX, 1 mM HEPES, and 1% penicillin/streptomycin) (29). The cells were fed with fresh medium every 2 days of culture. At day 10, macrophage purity was about 70%, as determined by flow cytometric analysis of the surface expression of macrophage markers CD11b and F4/80 using a LSR-Fortessa (BD Biosciences).

### Co-Culture of BMDMs and Exosomes

At day 10 of BMDM culture, cells were treated with 10 µg of exosomes for 24 h. Control cells were treated with either an equivalent particle number of 100-nm liposomes (Encapsula Nanoscience) as determined using TRPS or PBS alone. Exosome uptake was inhibited by incubation of BMDMs with 5 µM EDTA (Sigma-Aldrich) for 1 h before exosome treatment. To inhibit exosomal gp130, exosomes were treated with *N*′-(7-Fluoropyrrolo[1,2-a]quinoxalin-4-yl)-2-pyrazinecarbohydrazide (SC144, Sigma-Aldrich) (30). Exosomes were incubated with SC144 for 1 h and were later re-purified using qEV size exclusion chromatography columns.

### Flow Cytometry Analysis

BMDMs were detached from culture plates using cold PBS. Cell suspensions were stained with the respective antibodies (Table S1 in Supplementary Material), together with Fc-receptor blocking using anti-CD16/32, and washed with PBS containing 2% FBS. DiD-labeled exosome-positive cells were detected using red laser excitation and 640-nm emission. Flow cytometric acquisition was carried out on a LSR-Fortessa (BD Biosciences), as previously described (31). Data analysis was performed using FlowJo software (Tree Star).

### Immunofluorescence Microscopy

Immunofluorescence microscopy was performed as previously described (32). Briefly, BMDMs were seeded on cover slips and incubated with exosomes, liposomes, or PBS alone. Samples were fixed with paraformaldehyde for 1 h at room temperature. The cover slips were then incubated with primary antibodies and secondary, fluorochrome-conjugated antibodies. Cover slips were placed on slides containing ProLong^®^ Gold Antifade Mountant with DAPI liquid mountant (Life Technologies). Images were taken on a Zeiss 780-NLO confocal microscope with 40× and 100× magnifications.

### Analysis of IL-6 Secretion by Macrophages

Secretion of IL-6 by BMDMs was measured using Mouse IL-6 Quantikine ELISA Kit (R&D Systems) according to the manufacturer’s instructions.

### qRT-PCR

To analyze RNA expression levels on BMDMs, qRT-PCR was performed. Briefly, RNA was extracted by Trizol and cDNA synthesis was conducted using the SuperScript™ III First-Strand Synthesis system (Invitrogen Life Technologies), according to the manufacturer’s instructions. qRT-PCR was performed using Syber green master mix (Life Technologies). The data were analyzed using the 2^−ΔΔCt^ method and relative gene expression levels normalized to GAPDH. Primer sequences are detailed in Table S2 in Supplementary Material.

### Cell Survival Analysis

BMDMs were seeded on 6-well plates. Differentiated BMDMs were submitted to different treatments (without L cell supernatant), and the plates were placed in the IncuCyte live-cell imaging system (Essen Bioscience). Cell confluence (measured as the area of the field of view covered by cells) was assessed at 5 time points, as an average of 16 images captured per time point. Data were normalized to cell count at 0 h.

### Statistical Analysis

Data are presented as the mean ± SEM of results obtained from at least three independent experiments. Statistical significance was assessed using two-tailed Mann–Whitney *U* tests, with *p* < 0.05 considered statistically significant. *p < 0.05, **p < 0.01, and ***p < 0.001 are indicated in the figure legends.

## Results

### Characterization of Breast Cancer Exosomes

**Figure 1:**
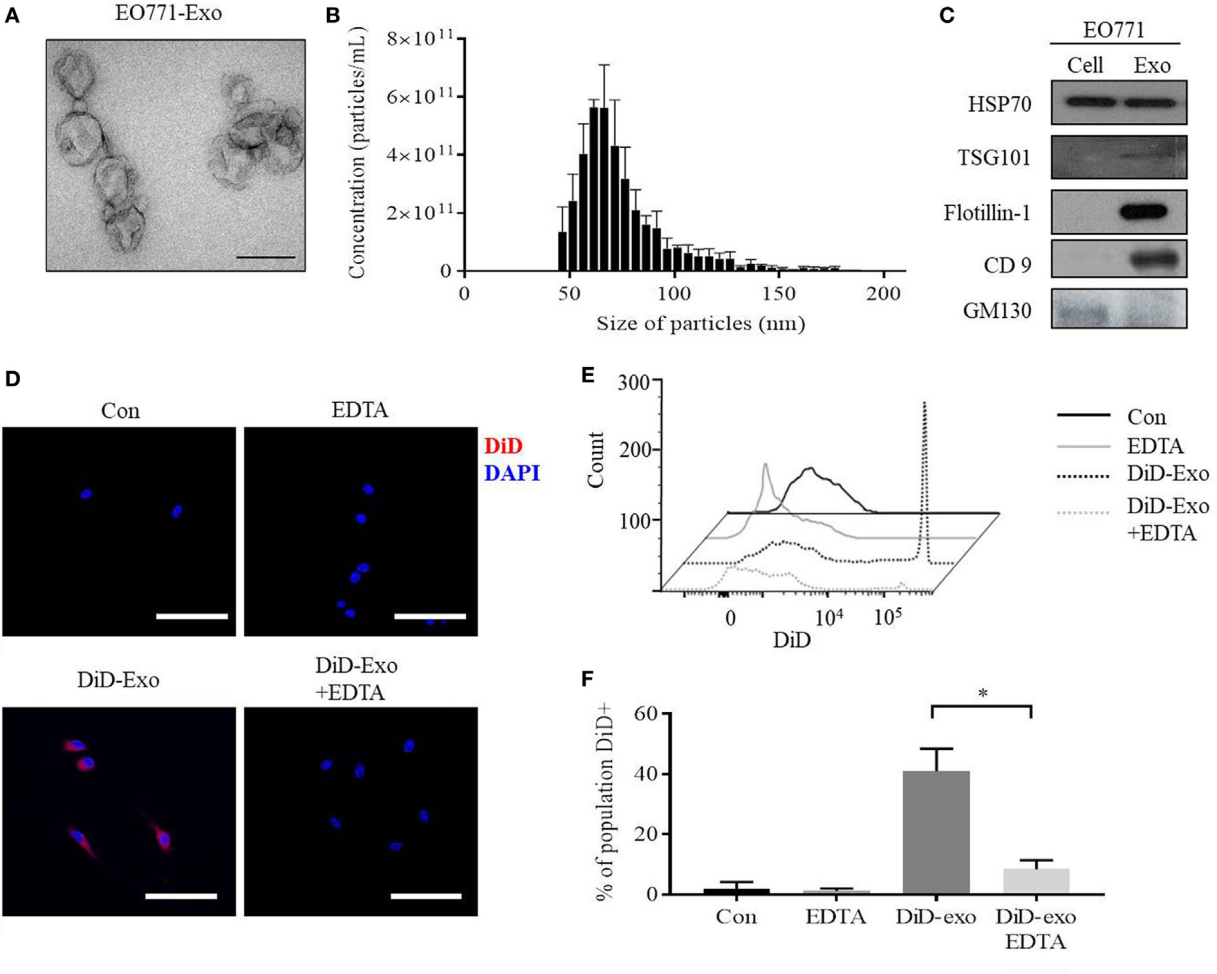
EO771 cells secrete exosomes which are taken up by bone marrow-derived macrophages (BMDMs). **(A)** Transmission electron microscopy of isolated particles indicates a sphere-shaped structure. The size bar represents 100 nm. **(B)** Size distribution and enumeration of particles assessed by Tunable Resistive Pulse Sensing (*n* = 3). **(C)** Expression of exosomal and cell markers in EO771 cell lysate (Cell) and exosomes (Exo). **(D)** Immunofluorescence imaging of DiD-labeled, EO771-derived exosome uptake into macrophages after 24 h. Macrophages were pretreated with EDTA (1 µM) for an hour as indicated. The size bar represents 50 µm. **(E,F)** BMDMs were gated for CD11b^+^/F4/80^+^ double-positive populations and DiD^+^ cells quantified. **(E)** Representative histogram of flow cytometry analyses. **(F)** Quantification of four independent repeats. *p < 0.05 as indicated.

The morphology of particles isolated from murine EO771 breast cancer cells is that of spherically shaped vesicles, with size ranging from 30 to 150 nm (Figures 1A,B). Furthermore, the particles are positive for the exosome marker proteins Tsg101, Flotillin-1, and CD9, but negative for the protein GM130 (Figure 1C). Collectively, these data show that the particles are exosomes as previously defined (33).

To assess if BMDMs are capable of taking up these exosomes, DiD-labeled exosomes were added to macrophage cultures. Macrophages acquire the DiD fluorophore after 24 h, indicating exosomal uptake (Figure 1D, lower left panel). EDTA has been shown to inhibit exosome uptake by interrupting calcium-dependent binding of exosomes to target cells (34). Indeed, EDTA is capable of reducing uptake of DiD-positive exosomes, while non-specific DiD dye uptake alone is not affected (Figure 1D, lower right panel; Figures S1A,B in Supplementary Material). Flow cytometry further confirmed that approximately 40% of all CD11b^+^/F4/80^+^ macrophages are DiD-positive after exposure to DiD-exosomes, and this is reduced to 7% by EDTA treatment (Figures 1E,F). These results indicate that macrophages take up EO771-derived exosomes, which can be inhibited by EDTA.

### Breast Cancer-Derived Exosomes Transfer gp130 to Induce STAT3 Signaling and Phenotypic Changes in BMDMs

**Figure 2:**
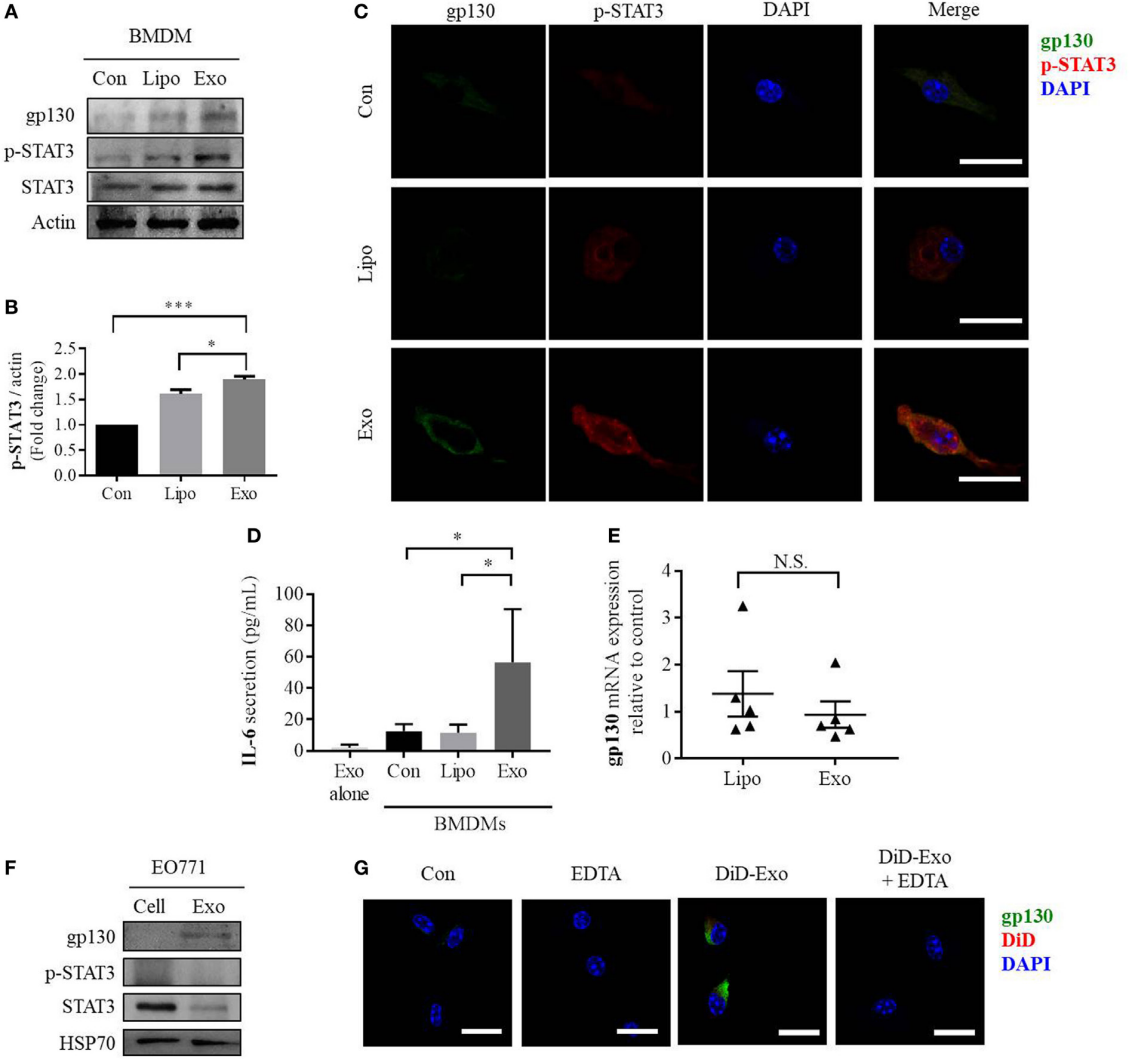
EO771-derived exosomes increase gp130 and p-STAT3 in recipient bone marrow-derived macrophages (BMDMs). **(A)** Macrophages were treated for 24 h with PBS alone (Con), liposomes (Lipo), or EO771-derived exosomes (Exo). Cell lysates were evaluated for gp130, p-STAT3, and STAT3 protein expression. Actin served as loading control. **(B)** Densitometry analysis of p-STAT3 levels as normalized by actin. **(C)** gp130 and p-STAT3 expression assessment after exposure of macrophages to PBS, liposomes, or EO771-derived exosomes for 24 h by immunofluorescence microscopy. The images were captured at 100× magnification and the size bar represents 20 µm. Nuclei of macrophages were visualized with DAPI. **(D)** Interleukin-6 (IL-6) secretion by macrophages after treatment with PBS, liposomes, or EO771-derived exosomes for 24 h was assessed by ELISA (*n* = 4). *p < 0.05 as indicated. As control, IL-6 protein content in exosomes is shown in the left column. **(E)** gp130 mRNA expression by macrophages after treatment with liposomes or EO771-derived exosomes for 24 h was assessed by qRT-PCR. Relative gene expression levels were normalized to GAPDH and results are shown as relative to PBS-treated BMDMs (*n* = 3). N.S., not statistically significant. **(F)** gp130, p-STAT3, and STAT3 protein expression in EO771 cell lysate (Cell) and exosomes (Exo). HSP70 is used as loading control. **(G)** Macrophages were pre-exposed to EDTA (EDTA) or PBS alone (Con) for an hour before treatment with DiD-labeled, EO771-derived exosomes for 24 h (DiD-Exo + EDTA and DiD-exo, respectively). DiD signal and gp130 localization were visualized by fluorescence microscopy. The size bar represents 20 µm.

Next, we evaluated the impact of cancer-derived exosomes on the IL-6/STAT3 signaling pathway in macrophages. After addition of exosomes, both gp130 and phosphorylated STAT3 levels increase compared with control groups of either PBS- or liposome-treated BMDMs (Figures 2A,B). Furthermore, phosphorylated STAT3 was found to translocate to the nucleus of BMDMs in response to incubation with cancer-derived exosomes (Figure 2C). Confocal immunofluorescence microscopy also showed that gp130 is localized to cell membranes of macrophages (Figure 2C). IL-6 is a key downstream target of the STAT3 signaling pathway and we observed an approximately three-fold induction in IL-6 secretion from BMDMs after cancer exosome exposure (Figure 2D). No IL-6 protein was found in cancer-derived exosomes alone (Figure 2D). Taken together, these data show that breast cancer-derived exosomes induce the gp130–STAT3 pathway, resulting in IL-6 secretion by BMDMs.

To determine the cause for the increase of gp130 and resulting STAT3 signaling in exosome-treated BMDMs, we evaluated the gene expression of gp130 in these cells. Surprisingly, there is no change in gp130 gene expression after incubation with exosomes (Figure 2E). This observation suggests potential extracellular sources for the elevated gp130 abundance. To explore if exosomal gp130 protein is causative for the increase in abundance of gp130 and IL-6 levels in BMDMs, the amount of gp130 and phosphorylated-STAT3 in cancer-derived exosomes and parental EO771 breast cancer cells was assessed. Specifically, gp130 was found to be enriched in the murine EO771 breast cancer cell-derived exosomes preparations (Figure 2F). Similarly, a range of human breast cancer cell line-derived exosomes (MDA-MB-231, MDA-MB-468, Hs578T, and MCF7) contained gp130 at various abundances (Figure S2 in Supplementary Material). To verify whether the accumulation of gp130 into BMDM cell membranes is indeed mediated by exosomes, we inhibited exosome uptake using EDTA and found that increased gp130 in BMDMs is attenuated by inhibiting exosome uptake (Figure 2G; Figure S3 in Supplementary Material). Collectively, these data suggest that exosomal gp130 protein could be transferred to BMDMs by cancer cell-derived exosomes and subsequently activate gp130–STAT3 signaling, thereby promoting IL-6 secretion.

**Figure 3:**
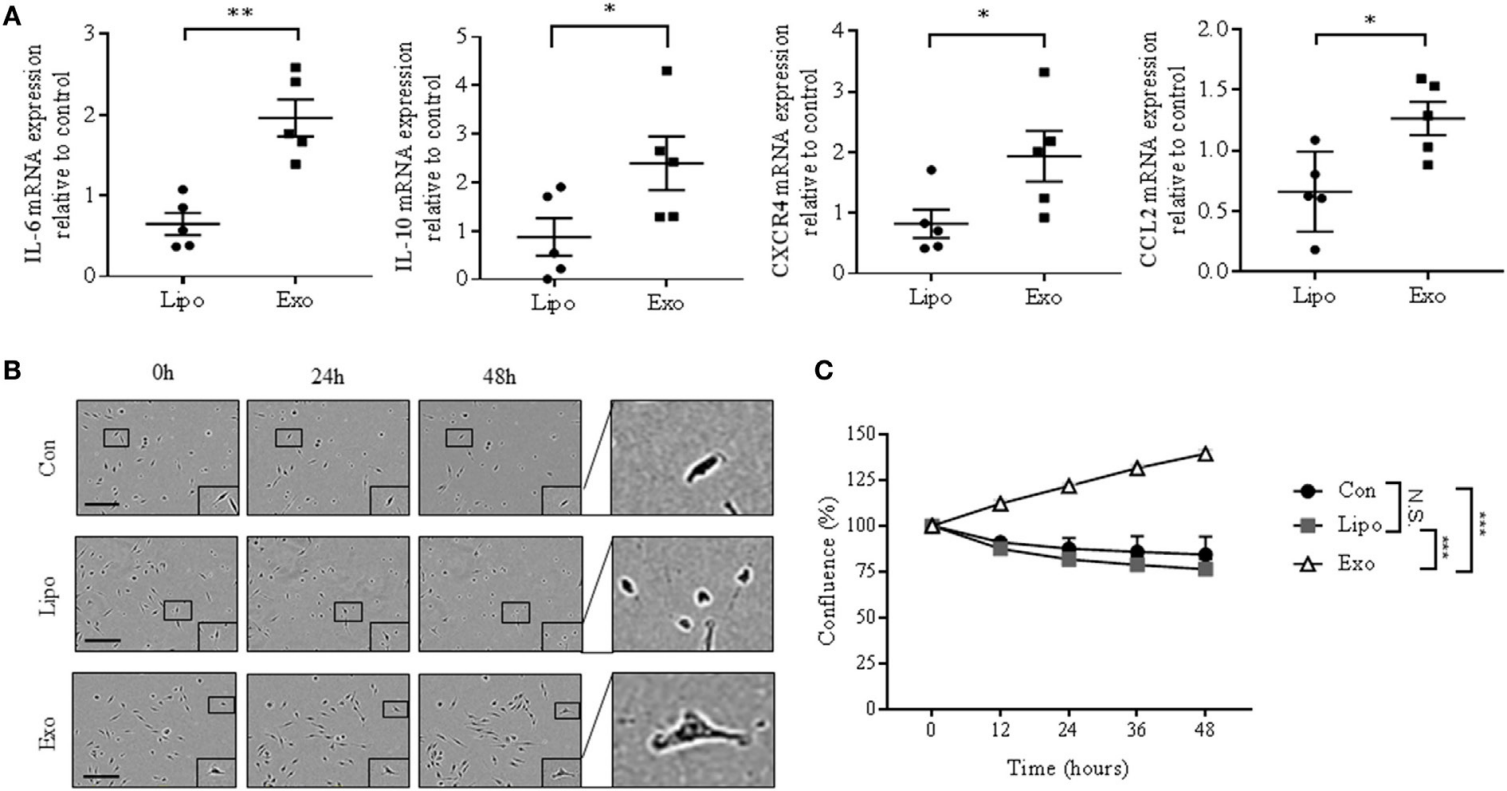
EO771-derived exosomes enhance mRNA expression of STAT3 target genes and survival rate of bone marrow-derived macrophages (BMDMs). **(A)** Interleukin-6 (IL-6), IL-10, CXCR4, and CCL2 mRNA expression was quantified by qRT-PCR in macrophages after liposome or exosome exposure for 24 h. Relative gene expression levels were normalized to GAPDH, and results are shown as relative to PBS-treated BMDMs (*n* = 5). *p < 0.05; **p < 0.01. **(B)** Incucyte images of macrophages after PBS, liposome, or exosome treatment for 0, 24, and 48 h. The size bar represents 150 µm. **(C)** Quantitative representation of Incucyte results. Cell confluence was assessed at 5 time points, as an average of 16 images captured per time point. Data were normalized to cell count at 0 h (*n* = 3 independent experiments). ***p < 0.001; N.S., not statistically significant, as indicated.

We next evaluated the impact of cancer exosomes on pro- and anti-inflammatory gene expression in BMDMs. After exosome exposure, mRNA levels of IFNγ, a M1 macrophage marker, significantly decrease compared with macrophages incubated with liposomes (Figure S4A in Supplementary Material). By contrast, IL-1β is upregulated, while other M1 markers, such as iNOS and TNFα, do not change (Figure S4A in Supplementary Material). Comparatively, Arg1 and TGF-β gene expression, which are indicative of M2 macrophage phenotype, are similarly not altered, whereas LOX gene expression is slightly elevated (Figure S4B in Supplementary Material). Together, these results suggest that cancer-derived exosomes alone are insufficient to generate a distinct M1 or M2 macrophage phenotype. Remarkably, IL-6, IL-10, CXCR4, and CCL2 mRNA, which are all STAT3 target genes involved in cancer progression (35, 36), were increased in macrophages exposed to exosomes (Figure 3A). STAT3 activation has also been associated with acquisition of malignant properties, such as increased cell survival (35). Exposing BMDMs to cancer exosomes resulted in an altered morphology and an increased survival of macrophages (Figures 3B,C). Taken together, our data indicate that breast cancer-derived exosomes induce phenotypical changes in macrophages, resulting in a pro-survival phenotype.

### Inhibition of Exosomal gp130 Reverses Cancer Exosome-Mediated Effects in Macrophages

**Figure 4:**
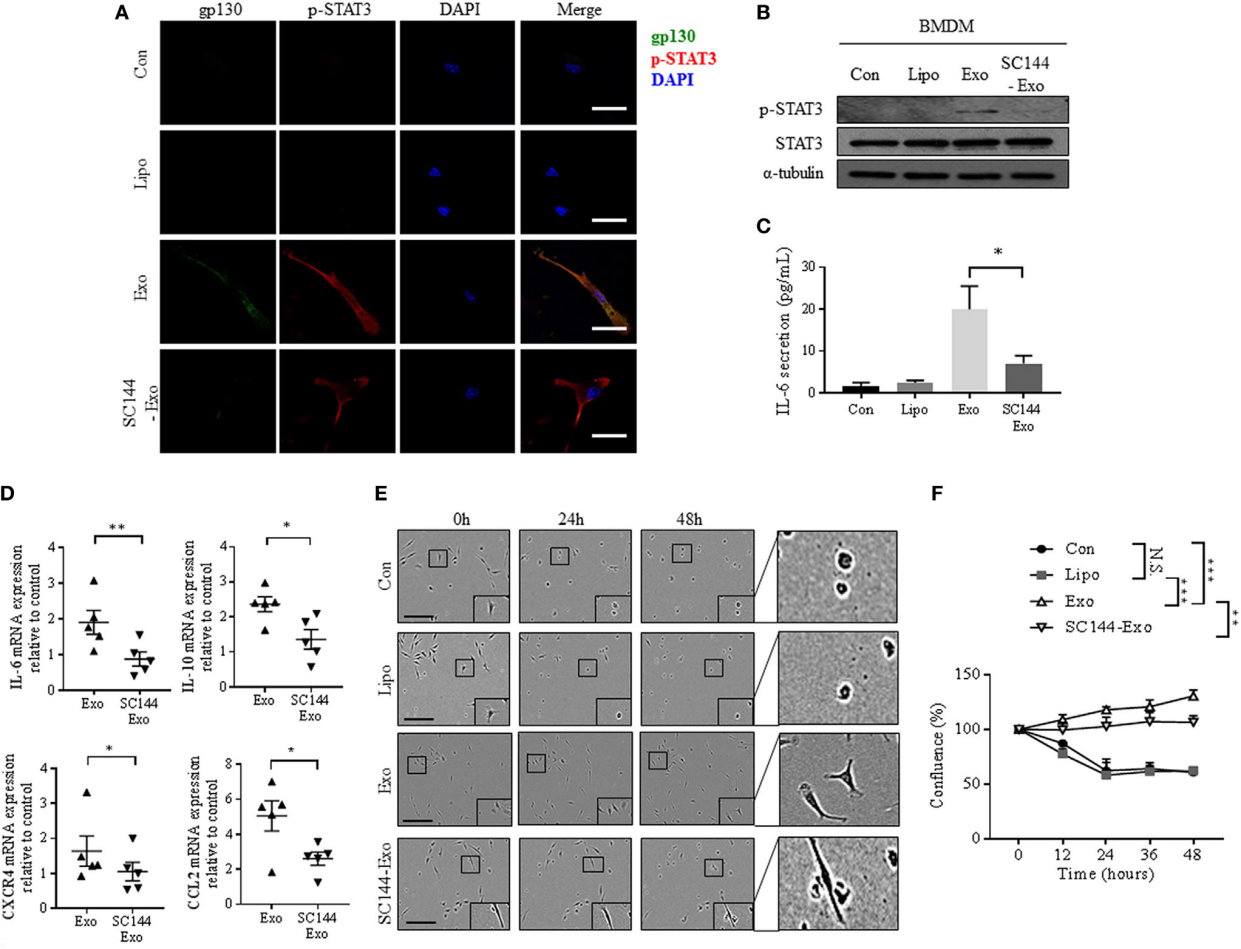
Inhibition of exosomal gp130 by SC144 reduces the breast cancer exosome-mediated phenotypical changes in bone marrow-derived macrophages (BMDMs). **(A)** gp130 and p-STAT3 expression were assessed after exposure of macrophages to PBS, liposomes, exosomes, or SC144 pretreated exosomes for 24 h by immunofluorescence microscopy. The images were captured at 100× magnification, and the size bar represents 20 µm. Nuclei of macrophages were visualized with DAPI. **(B)** The protein expression levels of p-STAT3 and STAT3 was assessed in macrophages cell lysates after the indicated 24-h treatments. α-Tubulin served as loading control. **(C)** Interleukin-6 (IL-6) secretion by macrophages after the indicated 24-h treatments was assessed by ELISA (*n* = 6). *p < 0.05 as indicated. **(D)** IL-6, IL-10, CXCR4, and CCL2 gene expression was quantified by qRT-PCR in macrophages after 24-h exposure to exosomes or SC144 pretreated exosomes as indicated. Relative gene expression levels were normalized to GAPDH and results are shown as relative to PBS control (*n* = 5). *p < 0.05; **p < 0.01. **(E)** Incucyte images of macrophages after indicated treatments for 0, 24, and 48 h. The size bar represents 20 µm. **(F)** Quantitative representation of Incucyte results. Cell confluence was quantified at 5 time points, as an average of 16 images captured per time point. Data were normalized to cell count at 0 h (*n* = 4 independent experiments). **p < 0.01; ***p < 0.001; N.S., not statistically significant, as indicated.

To confirm that transfer of exosomal gp130 is causative for the phenotypical changes in BMDMs, we incubated exosomes with SC144, a gp130 inhibitor (37). Pre-treatment of cancer exosomes with SC144 decreased both exosome-mediated phosphorylated STAT3 levels and nuclear translocalization in BMDMs (Figures 4A,B). In addition, BMDMs incubation with SC144-treated cancer-derived exosomes resulted in a reversal of the IL-6 secretion phenotype (Figure 4C). Similarly, exosome-mediated gp130/STAT3-induced gene expression was reduced when exosomes were pretreated with SC144 (Figure 4D). Finally, the morphological and pro-survival changes induced by cancer-derived exosomes in BMDMs were reverted by SC144-treated exosomes (Figures 4E,F). Together, these data verify that exosomal gp130 is indeed causative for the observed STAT3 signaling, IL-6 secretion, morphological changes, and enhanced survival of BMDMs in response to cancer-derived exosomes.

## Discussion

Despite numerous reports on tumor-promoting functions of cancer exosomes, our knowledge of their role in immune cell responses is limited. Immune surveillance is usually associated with anticancer properties (38). However, within cancer microenvironments, immune cells often display altered phenotypes capable of contributing to tumor progression, including promotion of tumor growth, migration, pre-metastasic niche formation, and metastasis (39, 40). For instance, classical pro-inflammatory macrophages generally have activated STAT1 signaling, whereas tumor-associated macrophages are known to activate STAT3 (19, 25, 41). Macrophages capable of infiltrating a tumor mass have also been shown to promote cancer progression and metastasis (42, 43).

The role cancer-derived exosomes play on the modulation of bone marrow mesenchymal stromal cells has been previously studied in a neuroblastoma model, and ERK1/2 described to control the level of IL-6 and IL-8/CXCL8 (44). In gastric cancer, macrophages were activated by cancer exosomes *via* the NF-κB pathway, thereby promoting cancer progression (45). Furthermore, it has been observed that human breast cancer cell-derived exosomes induce the secretion of IL-6, TNFα, and CCL2 from both human THP-1 and murine RAW macrophage cell lines *via* the toll-like receptor 2/NF-κB signaling pathway (46). Despite these and other examples, it is still unclear as to how macrophages are capable of triggering cancer initiation and progression, and how their phenotypical alterations are caused by exposure to tumor-derived exosomes. Our work suggests that exosomal gp130 is a key mediator in macrophage phenotype alterations. Overexpression of gp130 is found in diverse cancer types such as brain, bladder, colorectal, and breast cancer (37, 47). It has also been implicated as the main mediator of STAT3 activation in various breast cancer cell lines (48). We found gp130 to be contained in exosomes derived from a range of murine and human breast cancer cells (Figure 2F; Figure S2 in Supplementary Material). Interestingly, tetraspanin CD9, normally enriched in exosome membranes, has recently been reported to stabilize gp130, thereby facilitating activation of STAT3 signaling in glioma stem cells (49). In the context of macrophages, an imbalance of gp130 signaling has an impact on M2 macrophage polarization (50). This causative impact suggests that gp130 might have an important effect on polarization of tumor-associated macrophages. In addition, STAT3 activation, which is a key downstream pathway of gp130 activation in macrophages, is associated with angiogenesis (51) as well as myeloid cell accumulation in future metastatic microenvironments (52). STAT3 is also commonly activated in tumor-infiltrating macrophages (25, 41). Therefore, STAT3 activation in macrophages has been associated with a pro-tumoral macrophage phenotype, cancer progression, and poor patient outcome (19, 53).

Previous findings demonstrate that proteins packaged into exosomes can maintain their activity after exosome uptake by recipient cells (54, 55). It has been reported that tyrosine kinase receptors in exosomes are transferable to monocytes and capable of activating MAPK pathways, thereby promoting cell survival (54). Here, we show that transfer of exosomal gp130 causes STAT3 activation in macrophages and increases macrophages survival. Activated, phosphorylated STAT3 translocates to the nucleus and induces target gene transcription, including several genes associated with tumorigenesis, such as IL-6, IL-10, CXCR4, and CCL2 (41, 56). Tumors from triple-negative breast cancer patients are highly infiltrated by macrophages expressing, and secreting, both IL-6 and IL-10 (57). Each of these cytokines has specific roles in regulating the immune system and cancer surveillance. Secretion of IL-10 by macrophages results in immune-suppressive effects *via* dendritic cells and cytotoxic T cells modulation (58). Increased CXCR4-expressing macrophages were detected in the bone marrow of melanoma patients, which was associated with pro-angiogenic and immune-suppressive phenotypes (59). Moreover, IL-6 and CCL2 induce tumor-associated macrophage polarization (24, 26). Taken together, these data suggest that expression of the aforementioned pro-tumorigenic genes in macrophages could alter their phenotype toward a tumor-associated one. Finally, STAT3 signaling has been linked to cell survival (56). For example, it has been reported that STAT3 activation *via* gp130 in enterocytes is associated with cell survival signaling and cell cycle progression in the tumor microenvironment (60). Another study suggested that M2-like macrophages overexpressing anti-inflammatory cytokines can survive longer than M0 or M1 macrophages (61). These findings indicate that a long lifespan is one of the characteristics of tumor-associated macrophages.

To date, the commercially available inhibitor of IL-6 receptor, tocilizumab, and a gp130 specific inhibitor, FE999301, are only available for the treatment of autoimmune diseases, such as rheumatoid arthritis and inflammatory bowel disease. Despite both IL-6 receptor and gp130 also contributing to cancer progression (62), no IL-6 receptor antagonist is currently under clinical development in oncology. Recently, the blocking of IL-6/gp130/STAT3 has been suggested as anticancer drug approach (30, 63). One of these inhibitors, SC144, has been used to slow prostate, lung, breast, colorectal, and ovarian cancer progression and inhibit angiogenesis, in preclinical models (30, 64). SC144 is a small molecule inhibitor of gp130 and binds to S782 phosphorylated gp130, resulting in subsequent deglycosylation and inactivation of gp130 (30). Therefore, it abrogates downstream STAT3 phosphorylation and nuclear translocation (30). We show that exosomal gp130-induced effects are reversed when breast cancer-derived exosomes are pretreated with SC144. Together, these data are in agreement with the notion that inhibition of gp130 signaling could be an attractive therapeutic target in both breast cancer and other metastatic cancers (65, 66).

In conclusion, our data suggest that cancer-derived exosomal gp130 plays a critical role in the tumor environment *via* activation of the IL-6/STAT3 pathway in macrophages. This activation subsequently promotes BMDM survival and induces the expression of pro-tumorigenic cytokines, thereby potentially skewing BMDMs to a cancer-promoting phenotype. Although limited to a murine model, these results provide evidence demonstrating the role of exosomes in facilitating the exchange of cargo between cancer and immune cell subsets. The presence of gp130 in exosomes derived from human breast cancer cells, however, indicates that such mechanism of macrophage activation could operate in human cells as well. Altogether, this knowledge further improves our understanding of the implications of exosomal protein transfer in cancer progression.

## Ethics Statement

All animal procedures were conducted in accordance with Australian National Health and Medical Research regulations on the use and care of experimental animals, and approved by the QIMR Berghofer Medical Research Institute Animal Ethics Committee (A12617M, P1499).

## Author Contributions

AM, SH, LL, SW, and AW conceived the idea and designed the research. SH and SK harvested and maintained cells. RL conducted TRPS analysis. SH, LL, CE, and AM isolated exosomes and performed flow cytometry and western blotting. SH executed fluorescence microscopy and qRT-PCR. SH and AW performed and analyzed incucyte results. SH and AM wrote the manuscript. All the authors reviewed and approved the manuscript.

## Conflict of Interest Statement

The authors declare that the research was conducted in the absence of any commercial or financial relationships that could be construed as a potential conflict of interest.
