# Supplementary material for: Breast Cancer-Derived Exosomes Alter Macrophage Polarization via gp130/STAT3 Signaling
Source: Front Immunol. 2018 May 8;9:871. doi: 10.3389/fimmu.2018.00871 (PMC5951966; doi:10.3389/fimmu.2018.00871)
Supplement: Supplementary file 1 [file Data_Sheet_1.doc]

Supplementary Material

Breast cancer-derived exosomes alter macrophage polarization via gp130/STAT3 signaling

**AUTHORS**

Sunyoung Ham^1,2^, Luize G Lima^1^, Edna Pei Zhi Chai^1,3^, Richard J Lobb^1,3^, Sophie Krumeich^1^, Shu Wen Wen^4^, Adrian P Wiegmans^1^, Andreas Möller^1,2,3,*^

**AFFILITATIONS**

^1^ Tumour Microenvironment Laboratory, QIMR Berghofer Medical Research Institute, Herston, QLD 4006, Australia

^2^ School of Biomedical Sciences, Faculty of Health, Queensland University of Technology, Brisbane, QLD 4001, Australia

^3^ Faculty of Medicine, University of Queensland, Brisbane, QLD 4072, Australia

^4^ Centre for Inflammatory Diseases, Department of Medicine, School of Clinical Sciences, Monash University, Monash, VIC 3168 Australia

**^*^Correspondence:** Dr. Andreas Möller

Address: 300 Herston Road, Herston, QLD 4006, Australia, Tel: +61 7 3845 3950. E-mail: [andreas.moller@qimrberghofer.edu.au](mailto:andreas.moller@qimrberghofer.edu.au).

# Supplementary Figures


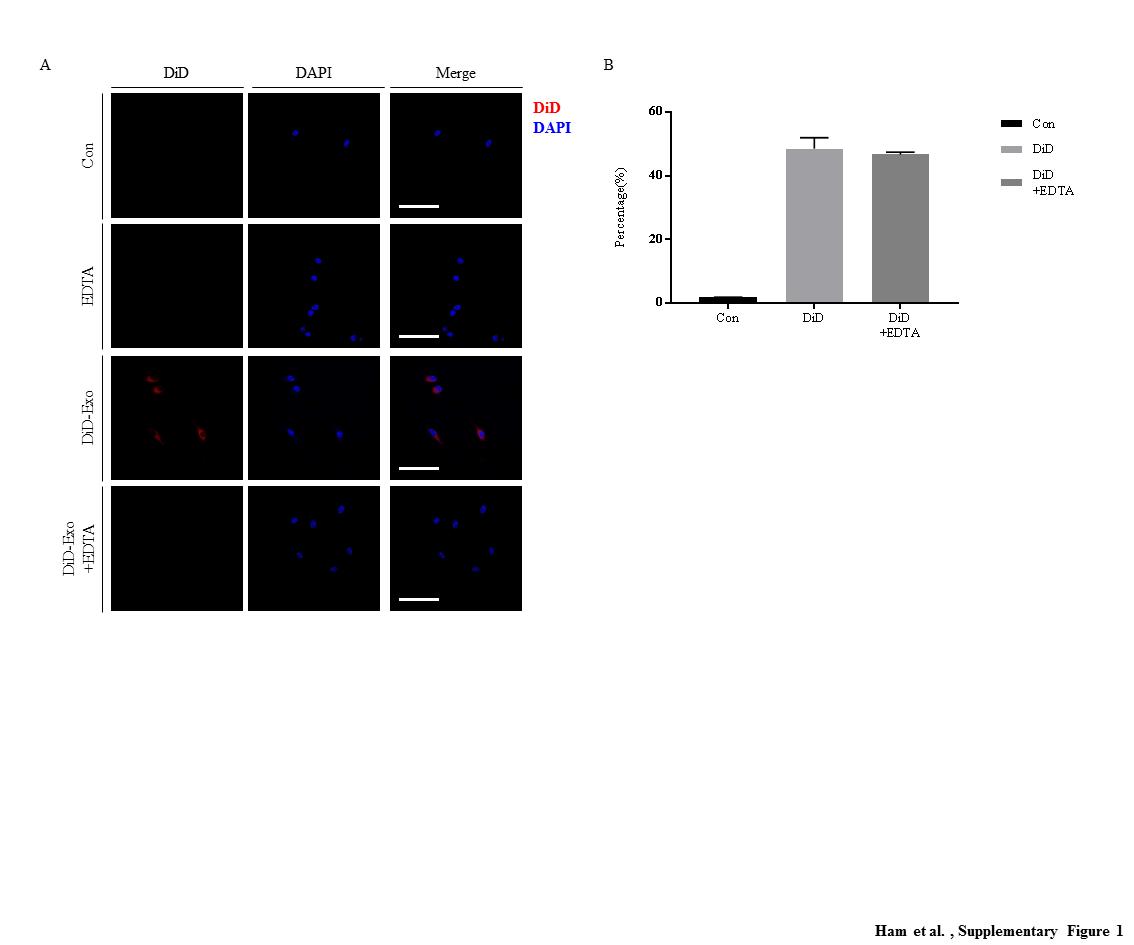


**Supplementary Figure 1.**

**(A)** Individual immunofluorescence images from Figure 1D. EO771-derived exosomes were labelled with DiD and nucleus were stained by DAPI. Macrophages were pretreated with EDTA (1μM) for 1 hour before treatment with DiD-labelled exosomes for 24 hours. The images were captured at 63x magnification; size bars indicate 50 μm. **(B)** BMDMs were pretreated with EDTA (1μM) for 1 hour before incubation with DiD alone for 24 hours. Cells were gated for CD11b^+^/F4/80^+^ double positive populations and the percentage of DiD^+^ cells quantified by flow cytometry. Quantification of 2 independent repeats is shown.


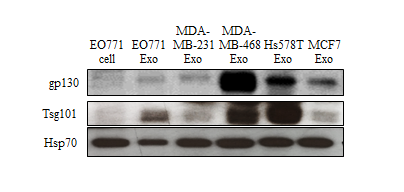


**Supplementary Figure 2.**

gp130 and Tsg101 protein expression in EO771 cell lysate and exosomes derived from different breast cancer cell lines. Hsp70 served as loading control.


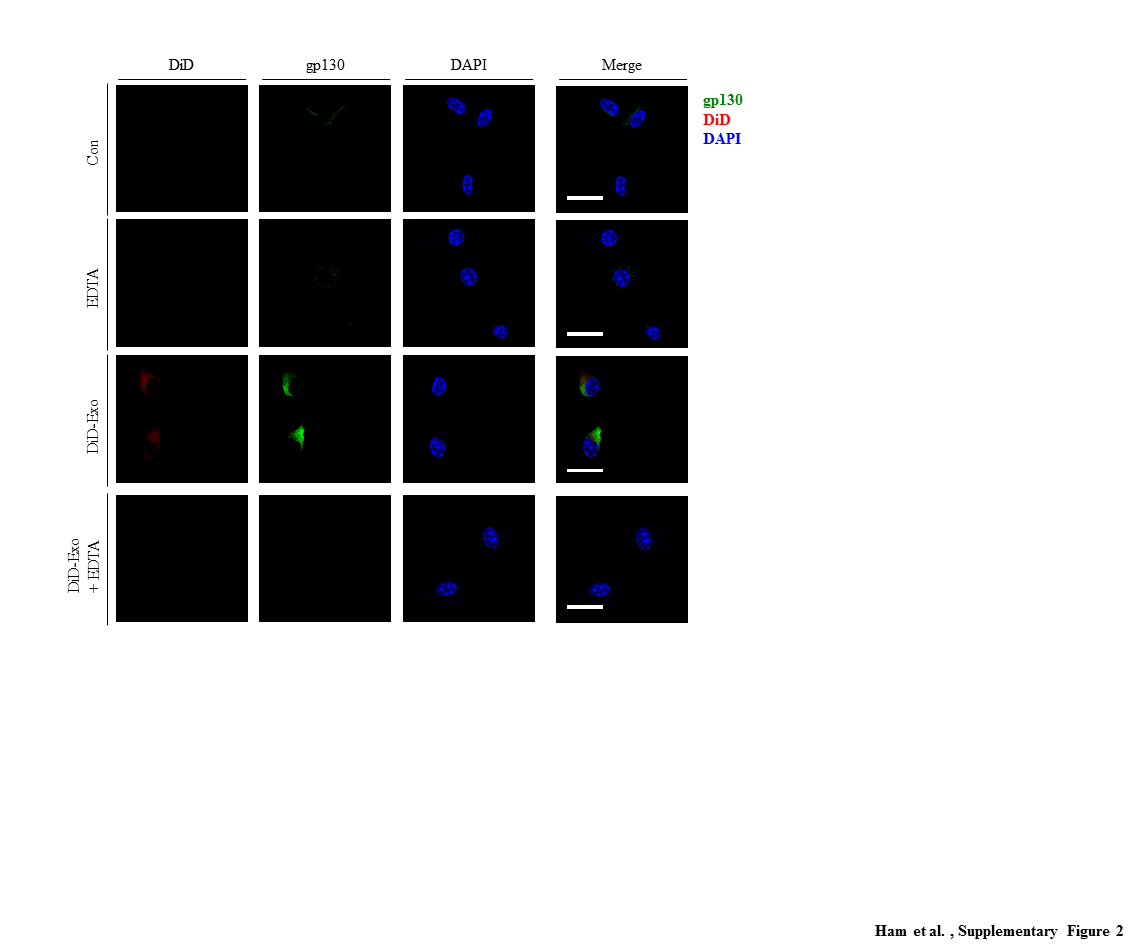


**Supplementary Figure 3.**

Individual immunofluorescence images from Figure 2F. Visualization of gp130 and DiD-labelled exosomes after pre-exposure of macrophages to EDTA (1μM) for 1 hour before treatment with DiD-labelled exosomes for 24 hours. The images were captured at 100x magnification and the size bars indicate 20 μm. Nuclei of macrophages were visualized with DAPI.


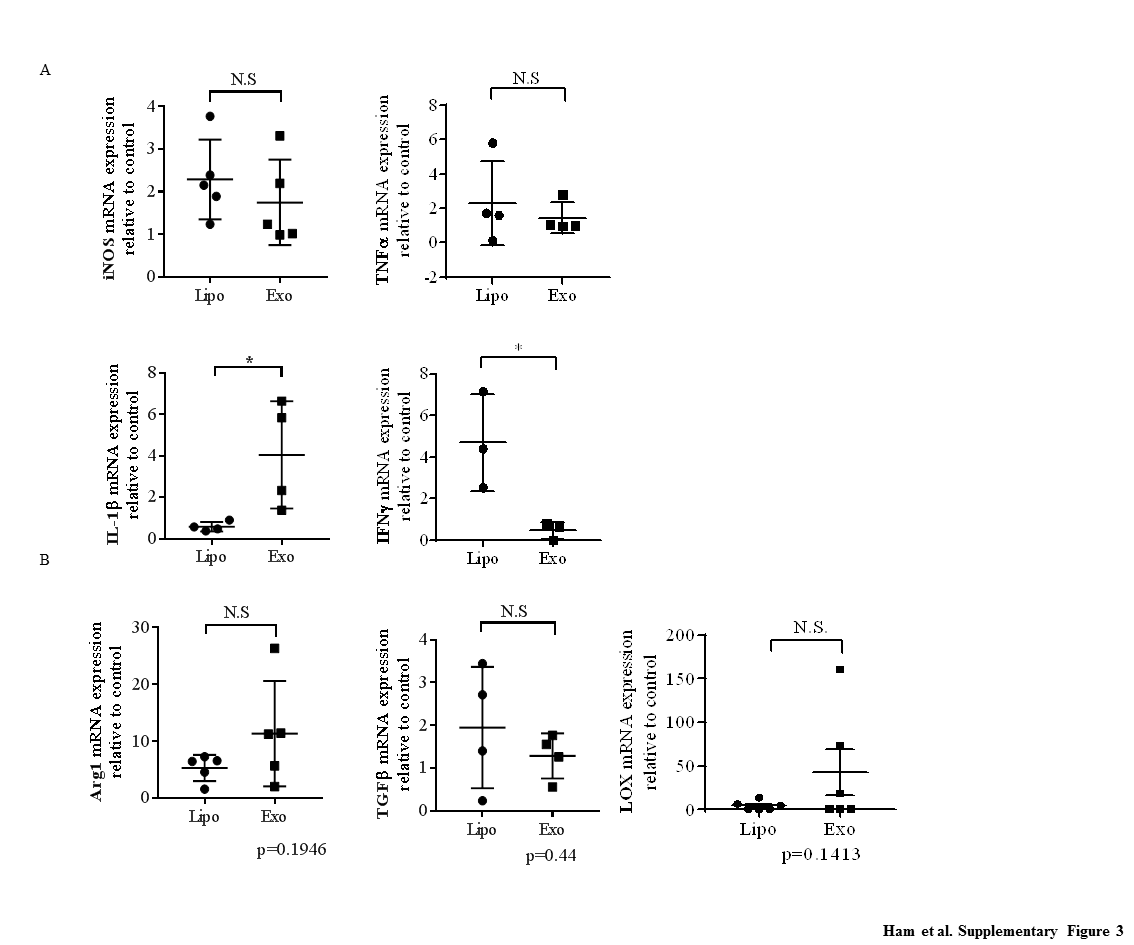


**Supplementary Figure 4.**

**(A/B)** mRNA expression of classical macrophage M1 (A: iNOS, TNFα, IL-1β and IFNγ) or M2 markers (B: Arg1 and TGF β) was assessed by qRT-PCR. Relative gene expression levels were normalized to GAPDH and results are shown as relative to PBS-treated BMDMs (at least n=3). *, p < 0.05; N.S., not statistically significant, as indicated.

**
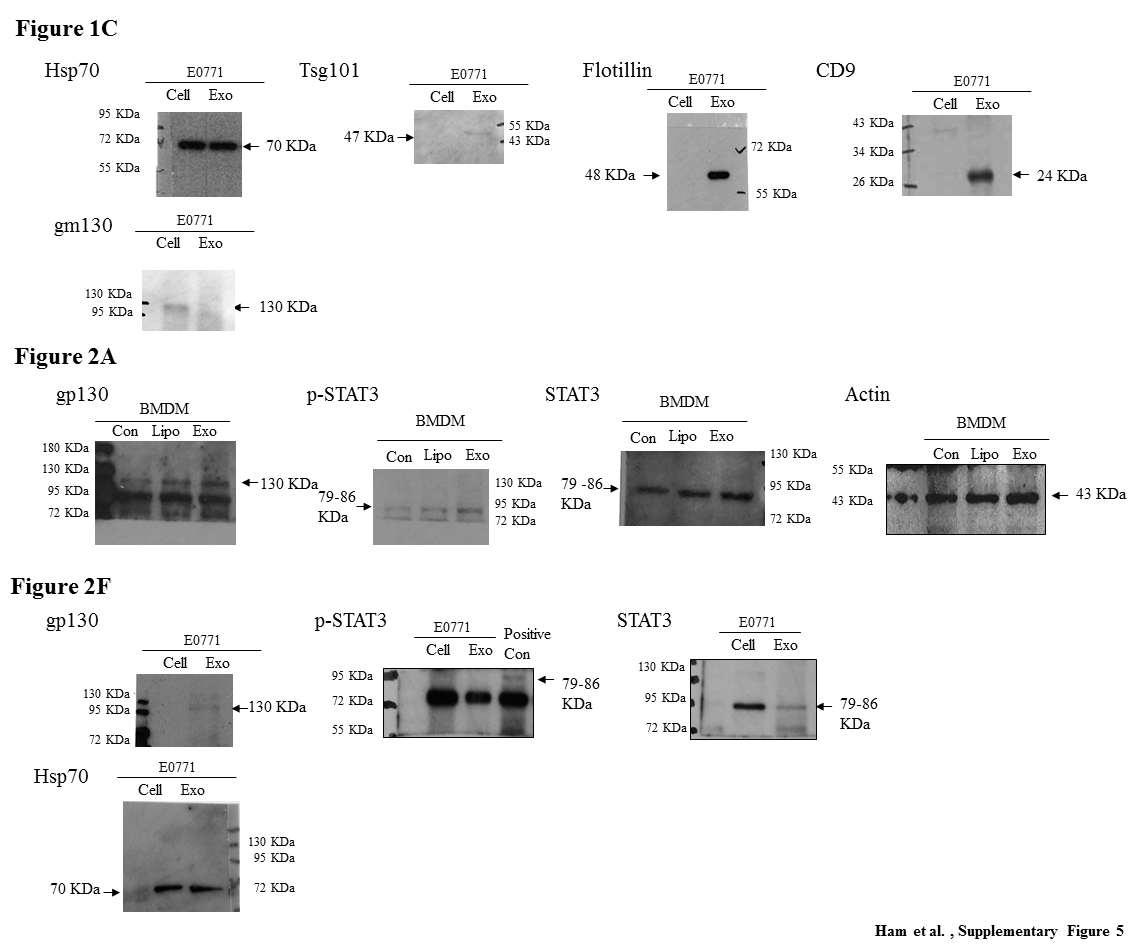
**

**
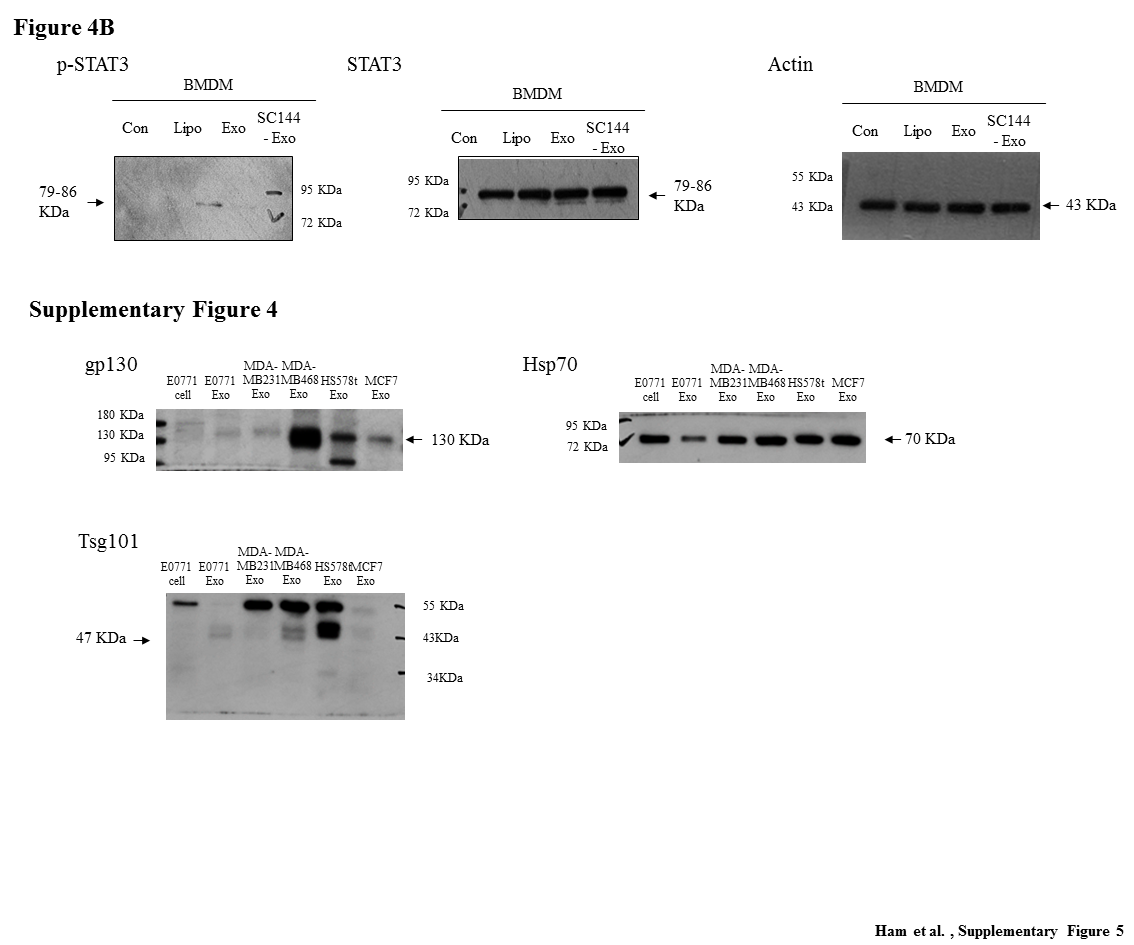
**

**Supplementary Figure 5.**

Images of full length western blots of each tested protein.

# Supplementary Tables


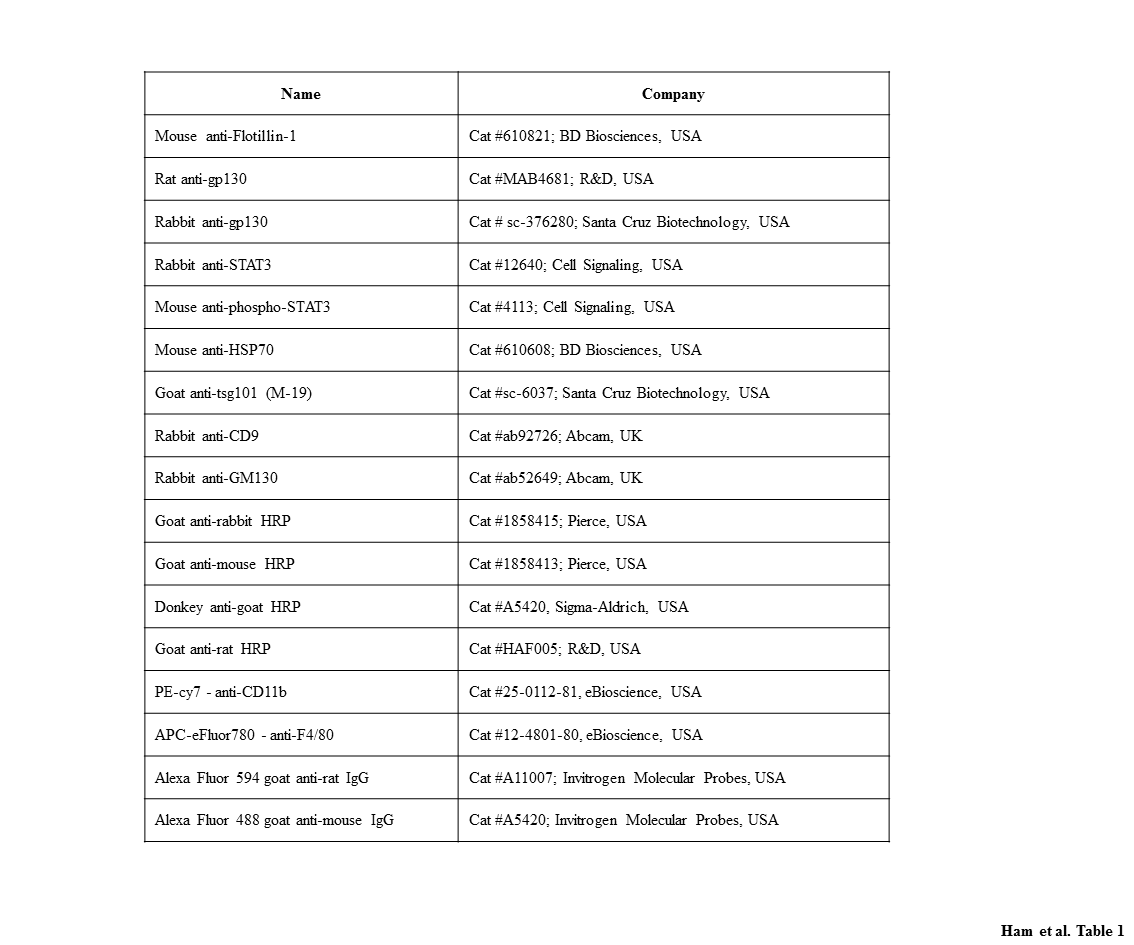


**Supplementary Table 1.**

List of antibodies applied for Western blotting, immunofluorescence and flow cytometry.


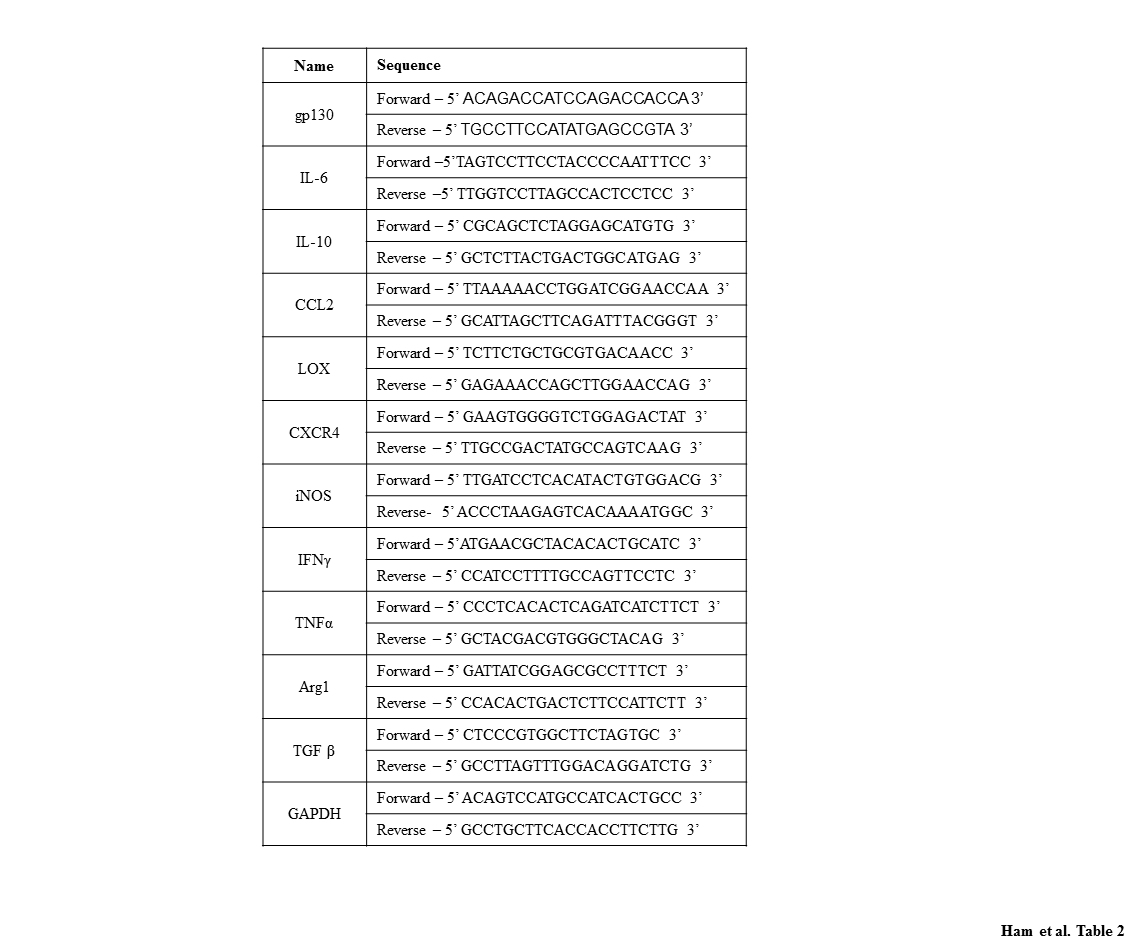


**Supplementary Table 2.**

List of primers applied for qRT-PCR.
